# Electrolyte Concentration in Urine and Urinary Infection—Is There Any Relation?

**DOI:** 10.3390/biomedicines13020253

**Published:** 2025-01-21

**Authors:** Ana Rita Ferrão, Paula Pestana, Lígia Borges, Rita Palmeira-de-Oliveira, Ana Palmeira-de-Oliveira, José Martinez-de-Oliveira

**Affiliations:** 1 Centro Hospitalar Universitário Cova da Beira EPE, Quinta do Alvito, 6200-251 Covilhã, Portugal; 2 RISE-Health, Department of Medical Sciences, Faculty of Health Sciences, University of Beira Interior, Av. Infante D. Henrique, 6200-506 Covilhã, Portugal; 3 Labfit–Health Products Research and Development Lda, Edifício Ubimedical, 6200-284 Covilhã, Portugal

**Keywords:** ions, urine, culture, Gram negative, infection

## Abstract

Background: Sodium, potassium, chloride, calcium, and magnesium in urine are useful biomarkers and are commonly evaluated in patients with different conditions. Urinary tract infections are among the most common diseases worldwide. However, their treatment poses significant challenges, particularly in hospitals, primarily due to antibiotic resistance and recurrence. Objectives: To evaluate the relationship between ion concentrations in urine and the presence of infection. Methods: A total of 175 random urine samples were collected from patients who had a request for urine culture at the Cova da Beira University Hospital Centre in Portugal. In vitro contamination was also conducted, in which ten negative urine cultures were contaminated with an *Escherichia coli* strain to evaluate the direct effect of its presence on the concentration of the ions. Results: In total, 61 samples were found to be positive, following a consensual quantitative definition. For Ca, there was a significant association between its concentrations in positive and negative cultures. In ten negative urine cultures experimentally contaminated with an *Escherichia coli* strain, bacterial growth did not seem to affect the concentration of ions. In vitro contaminated samples were also inoculated on MacConkey agar and incubated. The results showed that Gram-negative bacteria do not seem to proliferate in environments with low Ca concentrations. Conclusions: The presence of higher concentrations of Ca may facilitate the multiplication of Gram-negative bacteria, which can potentially result in depletion of Ca in vivo to putatively potentiate an inflammatory response. The concentration of Na, K, Cl, and Mg does not seem to have any relationship with UTIs.

## 1. Introduction

Urinary tract infections (UTIs) are common diseases worldwide, with approximately 150 million cases per year, making them one of the leading causes of antibiotic prescriptions [1]. The morbidity burden associated with this disease is well known and it is assumed to be relevant, often resulting in high costs for healthcare systems [2]. Although they are considered to be easily treated, they can cause serious complications and present challenges in treatment, especially in hospital settings [1].

They can occur in the first year of life, especially due to urethral malformations, but they are most common in females due to anatomical factors, such as the shorter urethra and the proximity with the anus and its normal gastrointestinal bacterial output [3]. There are also some life stages associated with a higher incidence of UTIs, in relation to sexual activity, pregnancy, and the menopause in women or with the existence of prostate diseases in men [4,5]. Currently, high rates of patients with recurrent UTIs and antibiotic-resistant bacteria are a big concern for specialists [6].

The most common pathogens responsible for UTIs are Enterobacteriaceae, especially *Escherichia coli* (*E. coli*) and *Klebsiella* spp., Pseudomonas aeruginosa, Enterococcus faecalis, Staphylococcus saprophyticus, and *Candida* spp. [1].

In clinical terms, UTIs are commonly classified as complicated or uncomplicated, the latter causing no additional problems, while the former are associated with other conditions, such as kidney stones, diabetes, immunosuppression, pregnancy, vesical catheterism, urinary tract abnormalities [1]. Pyelonephritis is a consequence of complicated UTIs and can lead to sepsis as a result of the ascending migration of pathogens [7]. Uncomplicated UTIs usually do not compromise superior urinary tract structures and do not cause fever. Cystitis is an uncomplicated UTI, typically presenting with symptoms such as dysuria, pollakiuria, urinary urgency, suprapubic pain, and sometimes hematuria [1,8].

Among the bacteria that cause UTIs, uropathogenic *E. coli* (UPEC) is responsible for 70% to 90% of those that are community acquired and 50% of nosocomial UTIs [1,9].

Although the symptoms of a UTI can be highly suggestive, a definite diagnosis is obtained when the number of microorganisms in urine after growth in artificial media (urine culture) is high. Urine must be collected under aseptic conditions, and the midstream of the first urine in the morning should be preferentially collected or at least 2 h after the last micturition [10,11]. Bacteriuria is defined as significant when there is growth of 100,000 colony-forming units (CFUs) per mL, although lower values can be considered clinically relevant in some cases such as, for example, the persistence of symptoms or if the urine has been collected through suprapubic bladder puncture [12]. The presence of leukocytes and pyocytes in a urinary analysis is also a useful predictor for UTIs, as well as nitrites which are common in the presence of *Enterobacteriaceae* [10,12].

Antibiotic resistance, the ability of bacteria to resist antibiotics that were once effective against them, poses serious problems in eliminating the infection [6].

Antibiotic resistance is a major health problem all over the world and, according to the European Centre for Disease Control (ECDC), it causes about 33,000 deaths per year in Europe, especially in the hospital context [13]. It is estimated that by the year 2050, antibiotic resistance will be responsible for about 10 million deaths per year. Incorrect and unnecessary use of antibiotics is the major cause of the appearance of resistant bacteria, which leads to serious clinical and economic consequences [14].

*Staphylococcus aureus* (*S. aureus*) and the genus *Enterococcus* are the most common antibiotic-resistant Gram-positive microorganisms, and *Acinetobacter baumannii*, *Pseudomonas aeruginosa,* and the family *Enterobacteriaceae* are the most frequent antibiotic-resistant Gram-negative microorganisms [6,7]. Extended-spectrum β-lactamase (ESBL)-producing bacteria are a big concern to antibiotic treatment, as some *Enterobacteriaceae* are becoming more and more resistant to various penicillins and cephalosporins. The resistance patterns also change from region to region [6].

Intake of antibiotics can promote side effects in the host organism, most commonly an alteration of the intestinal microbiota balance. This happens because most antibiotics enter the body by oral intake, and they can kill some of the normal and protective bacteria when reaching the intestine [15]. This can reduce the competition for space between normal and pathogenic microorganisms, sometimes leading to the proliferation of yeast fungi or non-sensitive bacteria. This imbalance of the normal flora may also occur in the vaginal environment, where *Lactobacilus* spp. usually play a protective role [16,17].

Regarding uncomplicated UTIs, around 22% of treated women develop vaginal candidiasis, which can occasionally cause an infection [18].

Treatment recommendations for UTIs may vary from country to country, according to the most common strains and their resistance patterns.

Currently, UTIs are treated empirically, with broad-spectrum antibiotics. This is due to the long time required for the results of the urine culture, which allows for the identification of bacteria after incubation and their antibiotic susceptibility tests [12,18]. For uncomplicated UTIs, Nitrofurantoin, Cotrimoxazole, and Fosfomycin are commonly recommended as empirical therapeutics. As alternatives fluoroquinolones (ofloxacin, ciprofloxacin, and levofloxacin) are proposed but side effects are more common. Because resistance rates are increasing all over the world, there are countries where oral fluoroquinolones are no longer recommended for empirical treatment. Β-lactams should be used carefully due to more adverse effects and less efficacy. Amoxicillin and ampicillin must not be used as empirical therapeutics [19].

Some patients do present with recurrent UTIs, which represents a challenge for treatment. UTIs are recurrent when they happen two or more times within 6 months or 3 or more times within 1 year. Many authors argue that it is necessary to reconsider empirical treatment or explore new therapeutic alternatives due to the increase in resistance rates and those of recurrence [2,20].

Phytotherapy has been proposed as a possible promising alternative treatment for UTIs or as a prophylactic for recurrent conditions. In this field, the species *Vaccinium macrocarpon* L. (cranberry) has been pointed out as an option [21]. The concentration of fructose and compounds such as anthocyanidin and proanthocyanidins in this species appear to interfere with the adherence of some bacteria such as *E. coli* to the uroepithelium. According to the type of fimbriae proteins present in the bacteria, these compounds can hypothetically bind to them, inhibiting their binding to the exposed lectins from the uroepithelium. Another hypothesis is that some of those compounds can probably affect fimbriae shape and make it difficult for them to bind to the tissue [22]. Other prophylactic therapies, such as probiotics and *E. coli* extracts, have been introduced to limit the increase of resistance rates to antibiotics [23].

Ions in urine are considered useful biomarkers for the diagnosis of several diseases and for their therapeutic monitoring. Among those, the determination of sodium (Na), potassium (K), chloride (Cl), calcium (Ca), and magnesium (Mg) in urine are the most commonly prescribed [19,24,25]. The source of ions is mainly represented by food and water intake, with them being absorbed in the gastrointestinal system and excreted mainly in the urine. Variations in the values for those ions in human urine are well described, as they are mostly associated with kidney problems and urinary malfunctions, among many other conditions [19].

To determine and monitor urinary levels of ions in patients, there are a variety of laboratory tests available, and nowadays, the most used is potentiometry automatized assays, which are simple to use and with very fast response times [26].

The concentration of K and Na do influence the permeability of cell membranes [27,28] and thus the uroepithelium. On the other hand, higher levels of Mg have shown to be good in cases of hyperactive bladder, as this ion has an important role in muscular function [29,30,31]. As occurs with Mg, levels of calcium may also interfere with muscular cells and proper functioning, but it has also been related to bacterial adhesion to cells in vitro [32,33,34]. These facts lead us to question whether ion concentration may influence bacterial proliferation or not.

This study aims to determine whether there is a relationship between the main ions in urine (Na, K, Cl, Mg, and Ca) and the existence of a UTI. If such a relationship exists, the objective of this investigation relies on finding out in which direction it occurs and if those results may have any importance in finding new approaches for UTI prophylaxis.

## 2. Materials and Methods

For this study, 175 random surplus urine samples (which would have been discarded) were selected from a population of adult patients with urine culture prescriptions at the clinical pathology service of the Cova da Beira University Hospital Centre. For 6 months, samples were randomly selected from the microbiology laboratory once they were processed for bacterial culture testing. The clinical courses of these patients were consulted to obtain data such as sex, diagnosis, origin service, medication, and analytical results for urine culture for the characterization of the population. The information collected was registered using the IBM SPSS Statistics data editor. At this point, all data was codified, and the identity of the patients and the origin of the samples were removed. After collecting these data, each surplus urine sample was prepared in 2 aliquots for the determination of ions. For the determination of Mg and Ca, samples were acidified with chloride acid to a pH of 1 to avoid the precipitation of ammonium–magnesium phosphate or calcium salts. For the determination of K, Na, and Cl, aliquots of urine without chloride acid were used. All five ions were determined with the automated system COBAS^®^ 8000 Modular Analyser Series, an automated system that uses colorimetric and potentiometric methods for those determinations.

To verify if the presence and multiplication of bacteria could lead to an alteration of ion values in urine, a UTI was simulated in vitro in this study by contaminating some of the negative cultural surplus urine samples.

Ten negative urine cultures were used from the surplus urine samples and inoculated with *E.coli* (ATCC 25922) to a concentration of 0.5 McFarland each. The samples were then incubated at 35 °C (±2 °C) for 18 to 24 h, and the values of the ions in this study were determined and compared with their values before contamination.

On the other hand, to try to understand if different ion concentrations present in the urine samples affect bacterial growth or not, each one of the contaminated urine samples was also inoculated on MacConkey plate agar, with a calibrated loop of 1 µL for colony counting, and incubated for 24 h at 37 °C. Then, we observed whether the inoculated bacteria developed, and the results were compared with the concentration of ions.

This study was approved by the Health Ethics Committee of Beira Interior Hospital Centre, with the reference 70/2019, and thus, adherence to the Declaration of Helsinki was assured.

## 3. Results

In accordance with the patients’ clinical presentations, most of the urine culture results were negative, while 61 samples where positive with one isolated pathogen with at least 100,000 colonies per mL (Table 1).

**Figure 1:**
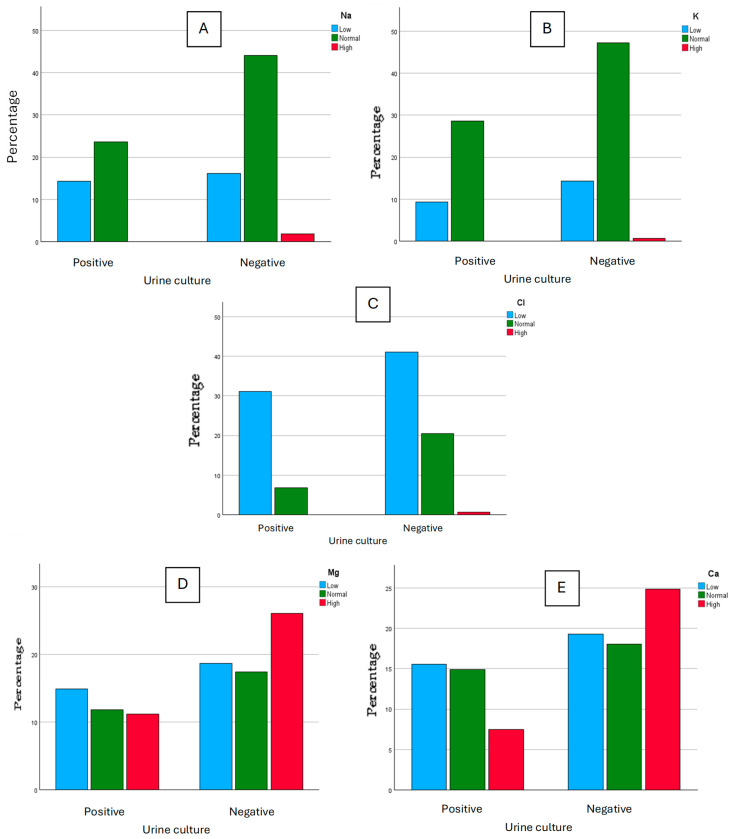
Graphical comparison for Na (**A**), K (**B**), Cl (**C**) (mmol/L), Mg (**D**), and Ca (**E**) (mg/dL) between positive and negative urine cultures.

Polymicrobial cultures were discarded from this study as those urine cultures were assumed to be contaminated during the collection process. The determination of Na, K, Cl, Mg, and Ca on each aliquot was performed, and the results were clustered into three classes for each ion, according to the reference values suggested on the reagent’s information sheets, as “low”, “normal”, and “high”. For Na, we considered as “high” the values >220 mmol, “normal” for values between 40 to 220 mmol, and “low” for values under 40 mmol. For K, we considered as “high” the values >125 mmol, “normal” for values between 25 to 125 mmol, and “low” for values under 25 mmol. For Cl, we considered as “high” the values >250 mmol, “normal” for values between 110 to 250 mmol, and “low” for values under 110 mmol. For Mg, we considered as “high” the values >5 mmol, “normal” for values between 3 to 5 mmol, and “low” for values under 3 mmol. For Ca, we considered as “high” the values >7.5 mmol, “normal” for values between 2.5 to 7.5 mmol, and “low” for values under 2.5 mmol. These results were then studied graphically and some differences between positive urine cultures and negative urine cultures seemed to appear (Figure 1). For Na, we obtained 23 “low” values for positive cultures and 26 “low” values for negative cultures, 38 “normal” values for positive cultures and 71 “normal” values for negative cultures, and 0 “high” values for positive cultures with 3 “high” values for negative cultures. For K, we obtained 15 “low” values for positive cultures and 23 “low” values for negative cultures, 46 “normal” values for positive cultures and 76 “normal” values for negative cultures, and 0 “high” values for positive cultures with 1 “high” value for negative cultures. For Cl, we obtained 50 “low” values for positive cultures and 66 “low” values for negative cultures, 11 “normal” values for positive cultures and 33 “normal” values for negative cultures, and 0 “high” values for positive cultures with 1 “high” value for negative cultures. For Mg, we obtained 24 “low” values for positive cultures and 30 “low” values for negative cultures, 19 “normal” values for positive cultures and 28 “normal” values for negative cultures, and 18 “high” values for positive cultures with 42 “high” values for negative cultures. For Ca, we obtained 25 “low” values for positive cultures and 31 “low” values for negative cultures, 24 “normal” values for positive cultures and 29 “normal” values for negative cultures, and 12 “high” values for positive cultures with 40 “high” values for negative cultures.

Within the graphical comparison regarding the ions Na, K, and Cl, there does not seem to be an evident difference in the classification of values between negative and positive urine cultures. However, there seems to be an association between the negativity of urine cultures and higher values of Mg and Ca.

To understand if there is any dependence between the results of urine cultures and ion concentration, a Chi-Square statistical test was performed, considering a confidence interval of 95%. According to the results, the urine cultures are independent of the classification of Na, K, Cl, and Mg values (*p*-values of 0.138, 0.722, 0.08, and 0.259, respectively). However, the urine culture results are dependent on Ca values (*p*-value of 0.028). Considering this association, the mean values of Ca for negative and positive urine cultures were considered (Table 2): in positive urine cultures, the mean value for Ca is significantly lower than with negative urine cultures.

**Figure 2:**
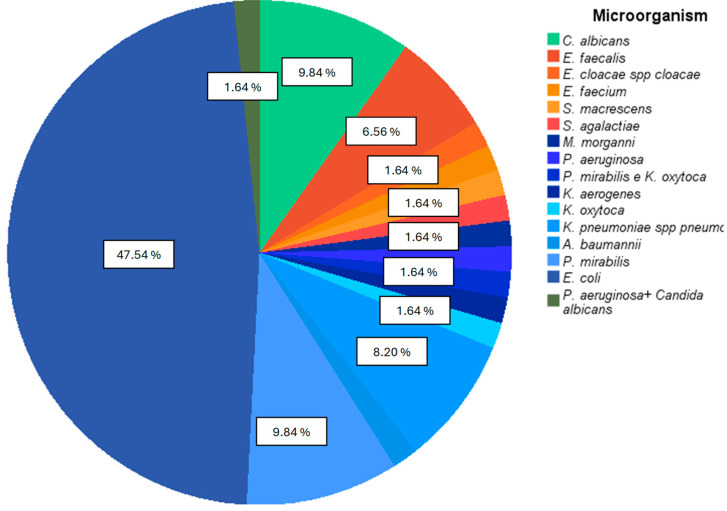
Percentage of each microorganism isolated in positive urine cultures. Gram-negative bacteria appear with bluish color sections, and Gram-positive bacteria appear with orange color sections.

Most of the pathogens isolated in positive urine cultures were Gram negative, mainly normal gastrointestinal bacteria (Figure 2), with *E. coli* representing the most isolated microorganism in positive urine cultures (47.54%)

As can be observed in Table 3, bacterial growth does not seem to modify the concentration of urine ions in vitro, in a 24 h window while bacterial growth develops, as the determinations at T0 (before contamination) and T1 (after contamination) are quite the same. However, this experience is not sufficient to discard the idea that bacterial growth in vitro can potentiate Ca influx and decrease Ca values in urine in vivo.

According to these results, there were two contaminated samples in which bacterial growth did not occur after inoculation on MacConkey agar and incubation. For these samples, numbers 4 and 9, the Na, K, and Cl values were significantly different from each other, while some of the positive results had ion concentrations within those ranges.

Regarding sample 4, it originally came from a patient that was taking antibiotics (amoxicillin plus clavulanic acid). However, it is interesting that in sample 9, the Ca values were much lower than the other samples and bacterial growth did not occur.

Images of the plates after inoculation with 24 h incubation at 37 °C are present in Supplementary Material S1.

## 4. Discussion

According to our statistical results, there is an association between the levels of Ca and the presence of a UTI. These results suggest that the presence of a UTI can either promote a decrease in Ca levels in urine or lower values of Ca in urine can promote bacterial multiplication and the development of a UTI.

One difficulty is that reference values for ions in urine are reported only for 24 h collections, which actually makes it difficult to say if the values obtained in an isolated sample are considered normal or not [26,35,36,37]. Reference ranges for 24 h collections are 40–220 mmol for Na, 25–125 mmol for K, 110–250 mmol for Cl, 3.0–5.0 mg/dL for Mg, and 2.5–7.5 mg/dL for Ca [35,36,37].

Lower values of urinary Na and Cl are usually related to significant water losses, as happens in some conditions like fever, diarrhoea, diabetes, respiratory infections, renal disease, and hypertension. Lower values of urinary Mg are commonly related to renal disease, and lower urinary Ca values are commonly related to decreased intake of calcium from the diet [19]. Many of the patients had base conditions that could cause loss of urinary Na, Cl, and Mg. On the other hand, Gram-negative bacteria tend to grow less in saline environments or with high concentrations of Mg [38,39,40].

Some authors have shown that the adhesion of bacteria to the epithelium can lead to an influx of Ca ions to potentiate the inflammatory response [41,42], and this may be the reason why our results showed lower Ca values in positive urine cultures. Furthermore, it has also been described how low concentrations of Ca in the environment seem to be associated with higher growth of Gram-negative bacteria [43].

Regarding the comparison of ion determination before (T0) and after contamination (T1) and the plate growth of in vitro contaminated urine cultures, in sample 4, as the antibiotic is a renally excreted compound, it is probably present in that sample, inhibiting bacterial multiplication even after in vitro contamination with *E. coli*. Concerning what happened with sample 9, it has already been observed that, when comparing surplus urine samples, Ca values are lower in positive urine cultures. This might give the impression that Gram-negative bacteria might need a certain amount of Ca to grow and are possibly not capable of multiplying in low Ca concentrations, but, if there is an availability of Ca they might multiply, and this could probably lead to a depletion of Ca in vivo in a urinary environment due to the influx of Ca to potentiate inflammatory response.

## 5. Conclusions

With this study, it is concluded that levels of Na, K, Cl, and Mg do not appear to interfere with bacterial growth and UTIs. However, there was an association between Ca levels and the presence of a UTI, suggesting that the presence of a UTI can either induce a decrease in Ca levels in urine or lower values of Ca in urine can promote bacterial multiplication and the development of a UTI.

To better evaluate ion concentrations in random urine samples, it is essential to establish reference ranges specifically for these types of samples rather than relying solely on 24 h collection samples.

Bacterial growth in vitro did not seem to alter ion concentrations in urine. Gram-negative bacteria need Ca to multiply in urine and cause a UTI. However, it seems that in vivo, their multiplication can cause a depletion of Ca, probably related to the activation of the inflammatory response, but further studies need to be performed to clarify this issue.

## Figures and Tables

**Table 1:** Urine culture results.

		Frequency	Percentage
Urine culture	Polymicrobial culture	17	9.7
	Positive	61	34.9
	Negative	97	55.4
	Total	175	100.0

**Table 2:** Means and standard deviations obtained for each ion with negative and positive urine cultures.

	Urine Culture	Mean	Standard Deviation
Potassium (mmol/L)	Positive	43.07	22.17
	Negative	48.65	28.14
Sodium (mmol/L)	Positive	64.66	44.71
	Negative	91.37	60.33
Chlorite (mmol/L)	Positive	63.43	45.44
	Negative	89.32	58.38
Magnesium (mg/dL)	Positive	4.13	2.93
	Negative	5.31	3.73
Calcium (mg/dL)	Positive	4.50	4.48
	Negative	7.47	7.15

**Table 3:** Comparison of ion determination before (T0) and after contamination (T1) and plate growth of in vitro contaminated urines.

Sample	Moment	Culture	Ions				
			Cl (mmol/L)	K (mmol/L)	Na (mmol/L)	Ca (mg/dL)	Mg (mg/dL)
**1**	T0	Negative	89.0	47.3	92.0	7.8	5.7
	T1		89.1	47.3	92.0	7.6	5.7
	Plate inoculation	Positive > 10^5^ CFU/mL					
**2**	T0	Negative	82.8	35.7	82.0	2.4	2.7
	T1		82.9	36.5	82.0	2.7	4.4
	Plate inoculation	Positive > 10^5^ CFU/mL					
**3**	T0	Negative	153.0	116.0	121.0	7.8	6.1
	T1		154.0	119.0	122.0	8.0	6.2
	Plate inoculation	Positive > 10^5^ CFU/mL					
**4**	T0	Negative	114.0	14.8	120.0	9.0	4.9
	T1		116.0	15.1	121.0	9.6	4.9
	Plate inoculation	Negative					
**5**	T0	Negative	101.0	48.3	102.0	17.9	7.3
	T1		103.0	51.4	99.0	18.2	7.5
	Plate inoculation	Positive > 10^5^ CFU/mL					
**6**	T0	Negative	29.3	108.0	42.0	7.0	5.7
	T1		31.0	113.0	38.0	7.4	5.6
	Plate inoculation	Positive > 10^5^ CFU/mL					
**7**	T0	Negative	128.0	23.1	138.0	7.4	4.7
	T1		130.0	23.8	136.0	7.6	5.0
	Plate inoculation	Positive > 10^5^ CFU/mL					
**8**	T0	Negative	153.0	90.2	135.0	6.2	7.6
	T1		156.0	96.8	135.0	6.5	7.7
	Plate inoculation	Positive > 10^5^ CFU/mL					
**9**	T0	Negative	69.7	121.0	47.0	0.6	5.0
	T1		68.9	128.0	42.0	0.5	4.7
	Plate inoculation	Negative					
**10**	T0	Negative	11.2	16.5	36.0	2.5	3.0
	T1		11.5	17.4	38.0	2.7	2.8
	Plate inoculation	Positive > 10^5^ CFU					

## Data Availability

The original contributions presented in this study are included in the article/Supplementary Material. Further inquiries can be directed to the corresponding author.

## Supplementary Materials

The following supporting information can be downloaded at: https://www.mdpi.com/article/10.3390/biomedicines13020253/s1. Supplementary material S1 (S1)—Plate images.

## Abbreviations

Na	Sodium
K	Potassium
Cl	Chloride
Ca	Calcium
Mg	Magnesium
UTI	Urinary tract infection
*E. coli*	*Escherichia coli*
UPEC	Uropathogenic *E. coli*
ECDC	European Centre for Disease Control
*S. aureus*	*Staphylococcus aureus*
ESBL	Extended-spectrum β-lactamase
