# Supplementary material for: Electrolyte Concentration in Urine and Urinary Infection—Is There Any Relation?
Source: Biomedicines. 2025 Jan 21;13(2):253. doi: 10.3390/biomedicines13020253 (PMC11851737; doi:10.3390/biomedicines13020253)
Supplement: Supplementary file 1 [file biomedicines-13-00253-s001.zip › biomedicines-3415384-supplementary.pdf]

Supplementary material S1 (S1)

Sample	Plate inoculation + 24h incubation 37ºC
**1**	Positive >10^5CFU/mL 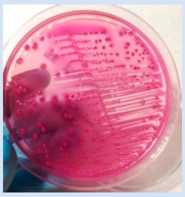
**2**	Positive >10^5CFU /mL 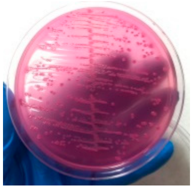
**3**	Positive >10^5 CFU /ml 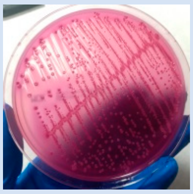
**4**	Negative 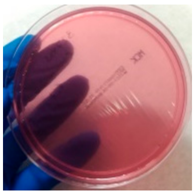
**5**	Positive >10^5CFU/mL 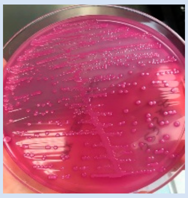
**6**	Positive >10^5CFU/mL 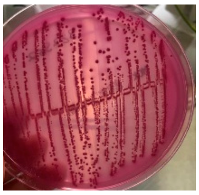

**7**	Positive >10^5CFU /mL 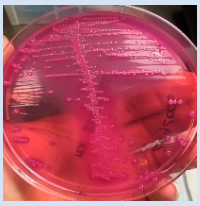
**8**	Positive >10^5 CFU/mL 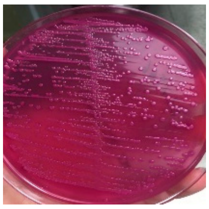
**9**	Negative 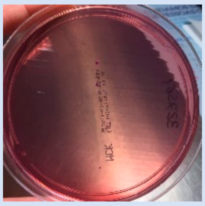
**10**	Positive >10^5 CFU 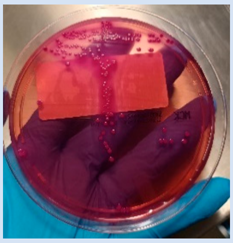
